# The complexity of human infected AIV H5N6 isolated from China

**DOI:** 10.1186/s12879-016-1932-1

**Published:** 2016-10-25

**Authors:** Zhijie Zhang, Rui Li, Lufang Jiang, Chenglong Xiong, Yue Chen, Genming Zhao, Qingwu Jiang

**Affiliations:** 1 Department of Epidemiology and Biostatistics, School of Public Health, Fudan University, Shanghai, People’s Republic of China; 2 Key Laboratory of Public Health Safety, Ministry of Education, Fudan University, Shanghai, People’s Republic of China; 3 Department of Public Health Microbiology, School of Public Health, Fudan University, Shanghai, People’s Republic of China; 4 School of Epidemiology, Public Health and Preventive Medicine, Faculty of Medicine, University of Ottawa, Ottawa, ON Canada; 5 Bldg. 8#, Rd. Dong’an 130, Shanghai, 200032 People’s Republic of China

**Keywords:** H5N6, Reassortment, H9N2, H5N1

## Abstract

**Background:**

Novel avian influenza viruses (AIVs) of H7N9, H10N8, and H5N6 are currently circulating in China’s poultry flocks, occasionally infecting human and other mammals. Human infected AIV H5N6 in China during 2014–2015 is believed to be a triple reassortant originated from H6N6 and two clades of H5 viruses. The current report suggests that its reassortment history is more complicated.

**Methods:**

Genomes of human infected isolates of AIV H5N6 were searched from the NCBI Influenza Virus Sequence Database and the Global Initiative on Sharing Avian Influenza Data. Sequences shared high identities with each segment of their genomes were obtained through the Basic Local Alignment Search Tool. Alignments were done by mafft-7.037-win32 program; 8 large-scale and then 8 gradually converged phylogenetic trees were constructed by using MEGA5.1/5.2/6.0 Software.

**Results:**

The events that each segment of the genomes of human infected AIV H5N6 isolates circulated in China had evolved into its current status might have happened before 2013, and so were they then reassorted into the epidemic AIV H5N6. A/Guangzhou/39715/2014(H5N6) and A/Sichuan/26221/2014(H5N6) had their six internal segments (PB2, PB1, PA, NP, NEP, and M) in common, and were reassorted from AIVs H5N1 in the same period and same region as that of HA, while A/Yunnan/0127/2015(H5N6) derived its six internal segments from AIV H9N2 that has been prevalent in Eastern China since 2008.

**Conclusions:**

AIV H5N6 isolates established from both human and poultry in China during 2014–2015 were heterogeneous; both AIVs H5N1 and H9N2 were involved in the reassortment of AIV H5N6 in China.

**Electronic supplementary material:**

The online version of this article (doi:10.1186/s12879-016-1932-1) contains supplementary material, which is available to authorized users.

## Background

Avian influenza viruses (AIVs) pose significant risks to public health; novel reassortants of AIVs H7N9, H10N8, and H5N6 are currently circulating in China’s poultry flocks, occasionally infecting human and other mammals [1–3]. Since a man in Sichuan province died of AIV H5N6 infection on May 6, 2014, which is the first known case of human infection of this flu subtype in the world [4], 9 confirmed human spillover infections of AIV H5N6 had been reported sporadically in China [5]. There are likely a lot more undetected H5N6 flu cases with no severe symptoms, especially in the children population [6]. To reduce the threat of human infections with novel or enzootic AIV subtypes, there is an urgent need to determine the factors, which contribute to the emergence of novel AIVs. We explored the origin of human infected AIV H5N6 through its reassortment history.

## Methods

Genomes of human infected isolates of AIV H5N6 were collected from the NCBI Influenza Virus Sequence Database (http://www.ncbi.nlm.nih.gov/genomes/FLU/aboutdatabase.html) and the Global Initiative on Sharing Avian Influenza Data (GISAID) database (http://platform.gisaid.org/epi3/frontend) on December 18, 2015. Sequences shared high identities with each segment of the genomes of these AIV H5N6 isolates were obtained through the Basic Local Alignment Search Tool (BLAST); the parameter of max target sequences was set as 1 000. The acquired matrices were merged into 8 new ones according to their coding proteins, and the repeated sequences in each of them were removed. Alignments were done by mafft-7.037-win32 program. We first applied the Neighbor-Joining statistical method, kimura 2-parameter model, and bootstrap test with 500 replicates to construct 8 large-scale phylogenetic trees by using MEGA 5.1/5.2/6.0 Software. Sequences that shared high pairwise identities and sited within the same branch were remained only the earliest one unless they were isolated from different countries or regions, since they might be the same strain obtained from different host individuals. After convergence, 8 relatively small matrices were used for further analyses; and then, the Jmodeltest 2 Program was used to determine the optimum nucleotide institution model to construct accuracy phylogenetic trees, where the maximum likelihood statistical method and bootstrap test with 1 000 replicates were applied. When analyzing internal 6 segments (PB2, PB1, PA, NP, NEP, and M), PanfluH1N12009 was served as the out-group; Novel AIVs H7N9 and H10N8 emerged in China during 2013–2014 also were discussed in this study.

## Results

Three genomes for human infected AIVs H5N6, A/Sichuan/26221/2014(H5N6), A/Yunnan/0127/2015(H5N6), and A/Guangzhou/39715/2014(H5N6) were obtained from the NCBI Flu Database and GISAID. And then, 8 segments, 24 matrices, and 24 000 sequences were obtained through the BLAST. After removing repeated sequences, the 8 merged large-scale matrices included 2 053, 2 037, 2 029, 1 075, 2 084, 1 040, 2 146, 2 062 sequences according to the segments PB2, PB1, PA, HA, NP, NA, M, NEP, respectively (Additional file 1: 1a–8a, and Additional file 2: 1b–8b). After convergence, 8 relatively small matrices consisted of 51, 51, 51, 28, 51, 35, 51, 51 sequences were obtained respectively; GTR + G + I was determined to be the optimum nucleotide institution model to construct accuracy phylogenetic trees.

**Fig. 1:**
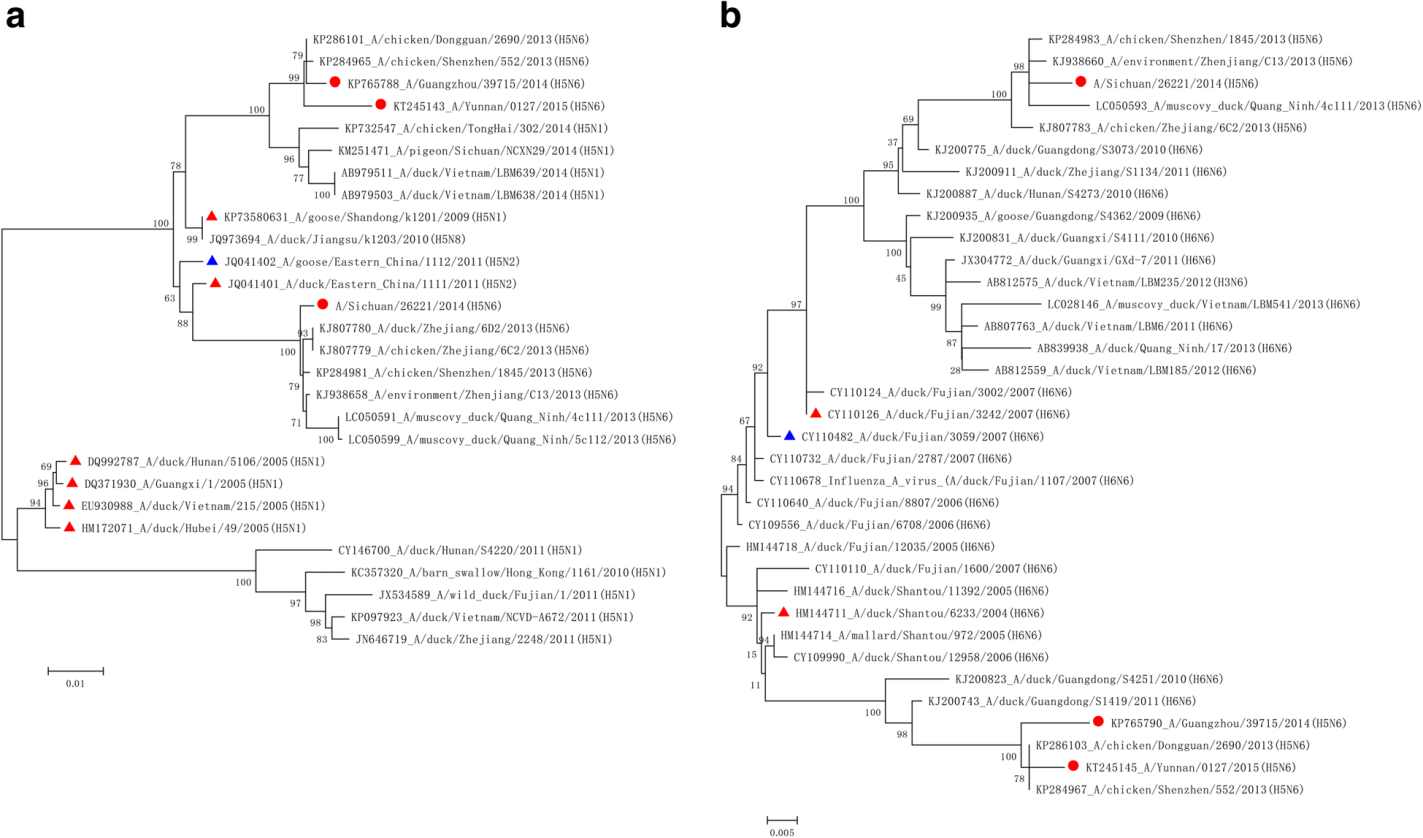
Phylogenetic trees of HA (**a**) and NA (**b**) genes of AIV H5N6 and reference isolates. It demonstrated that AIV H5N6 isolates obtained from both human and poultry in China were very heterogeneous. Sequences labeled with red circles are current human infected isolates of AIV H5N6, with red triangles are the sequences of probable ancestors based on the current study, and with blue triangles are sequences of probable ancestors based on opinions from other researchers

The data showed that each segment of the genomes of human infected isolates of AIV H5N6 circulated in China could have reached its current evolutionary status before 2013, and was then reassorted into the epidemic AIVs H5N6, which had infected poultries in China and South and Southeast Asia for a period of time before sporadical spillover infection to human beings. For HA, the three isolates of human derived AIV H5N6 might have AIV H5N1, which had circulated in China, and South and Southeast Asia since 2005 [e.g., A/Guangxi/1/2005(H5N1) or A/duck/Vietnam/215/2005(H5N1)], served as their remote ancestor. So far, the descendants of this AIV H5N1, including AIVs H5N1, H5N2, H5N6, and H5N8, have been prevalent in Eastern China and Southeast Asia such as Vietnam, Bangladesh, Laos, India, Nepal and others. For A/Yunnan/0127/2015(H5N6) and A/Guangzhou/39715/2014(H5N6), they probably descended from the same closer ancestor such as A/goose/Shandong/k1201/2009(H5N1) from Eastern China, while the closer ancestor of A/Sichuan/26221/2014(H5N6) is A/duck/Eastern_China/1111/2011(H5N2) (Fig. 1a).

The donor of NA for AIV H5N6 was AIV H6N6 that emerged before 2004. This H6N6 was frequently isolated in the coastal areas of South and Southeast China and evolved into two clades during 2004–2007; one clade [A/duck/Shantou/6233/2004(H6N6)] was reassorted into A/Yunnan/0127/2015(H5N6) and A/Guangzhou/39715/2014(H5N6) and the other clade [A/duck/Fujian/3242/2007(H6N6)] into A/Sichuan/26221/2014(H5N6) (Fig. 1b).

**Fig. 2:**
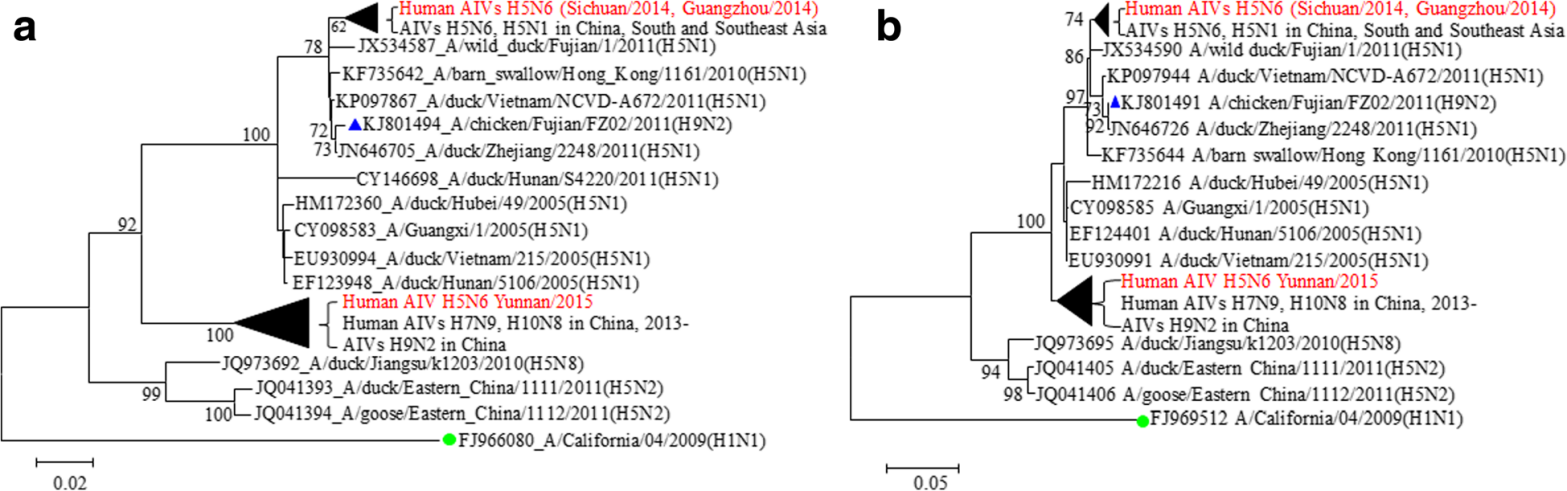
Phylogenetic trees of segments 1 (**a**) and 5 (**b**) of AIV H5N6 and reference isolates. In order to illustrate the reassortment characteristics of six internal segments of human infected AIV H5N6 in China, here takes the segment 1 and 5 as examples, which encode the proteins PB2 and NP of influenza virus, respectively. It displayed that not only AIV H5N1 circulated in China, South and Southeast Asia, but also the AIV H9N2, are involved in the reassortment of AIV H5N6 in China. Sequences in red fond are those ones which were discussed in this study; sequence labeled with green circle is of the PanfluH1N12009, which is taken as outgroup; with blue triangle is the sequence emphasized in this study

**Fig. 3:**
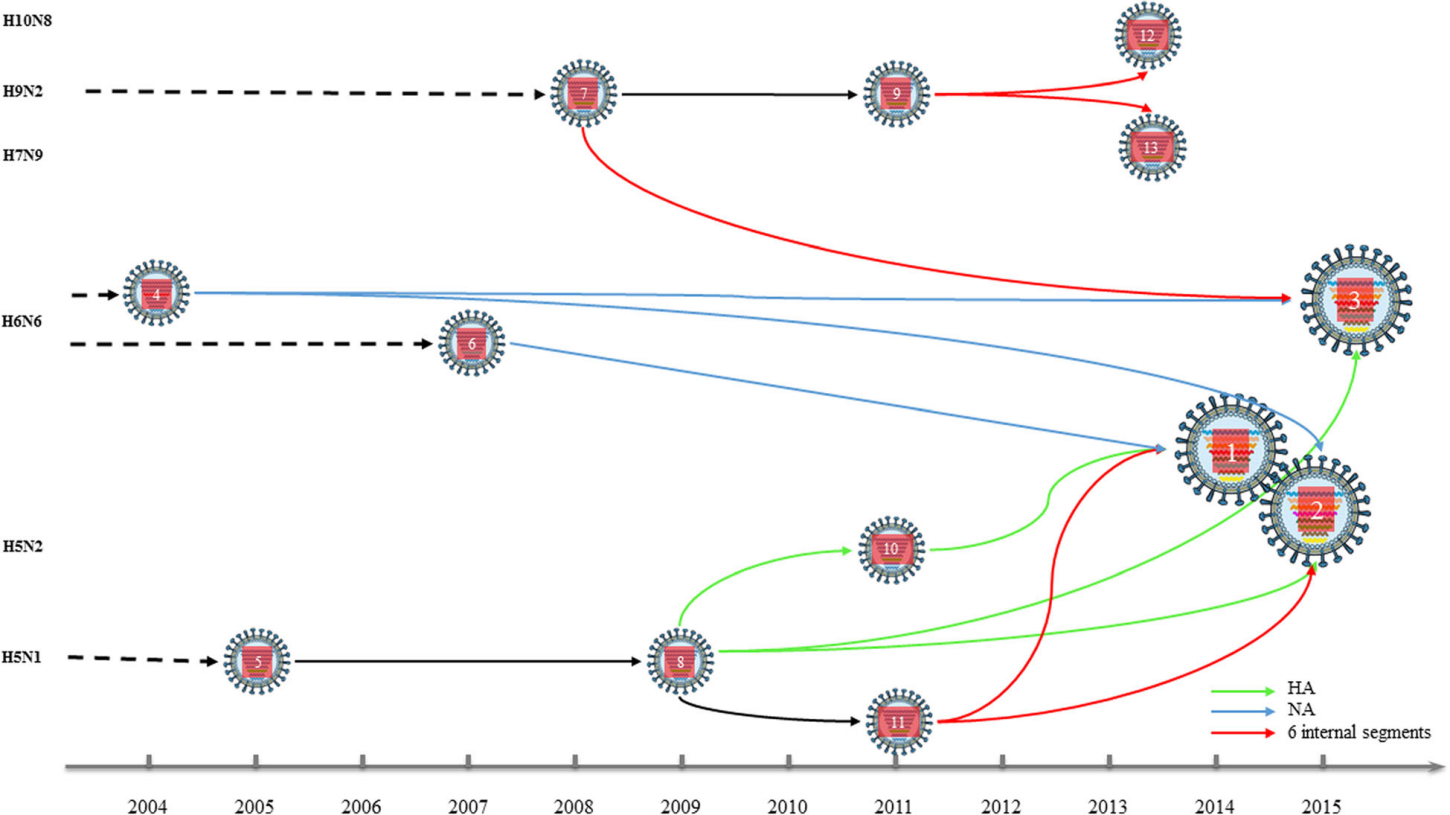
The probable reassortment history of human infected AIV H5N6 in China, 2014-2015*. 1, A/Sichuan/26221/2014(H5N6); 2, A/Guangzhou/39715/2014(H5N6); 3, A/Yunnan/0127/2015(H5N6); 4, A/duck/Shantou/6233/2004(H6N6); 5, A/Guangxi/1/2005(H5N1) or A/duck/Vietnam/215/2005(H5N1) and so on; 6, A/duck/Fujian/3242/2007(H6N6); 7, A/chicken/Hebei/DF/2008(H9N2); 8, A/goose/Shandong/k1201/2009(H5N1); 9, A/chicken/Jiangsu/B20/2011(H9N2); 10, A/duck/Eastern_China/1111/2011(H5N2); 11, A/wild duck/Fujian/1/2011(H5N1); 12, A/Jiangxi-Donghu/346-2/2013(H10N8); 13, A/Zhejiang/DTID-ZJU01/2013(H7N9). *Notes: (1) Each donor isolate represents one class of isolates in the same period, which was determined with the BLAST program on NCBI website and large-scale phylogenetic analysis (See online support materials); (2) Six internal segments of H10N8 and H7N9 in China since 2013 had common donors as AIV A/Yunnan/0127/2015(H5N6) in China

Six internal segments have more complex reassortment characteristics. A/Guangzhou/39715/2014(H5N6) and A/Sichuan/26221/2014(H5N6) have a similar origin from AIV H5N1 in the same period and region as that of HA, but their closer ancestor is A/wild duck/Fujian/1/2011(H5N1), which reassorted into the two isolates of AIV H5N6 around 2013, causing an epidemic in the poultry of China, and South and Southeast Asia. While the six internal segments of A/Yunnan/0127/2015(H5N6) is reassorted from AIV H9N2 that had been prevalent in Eastern China since 2008, named as A/chicken/Hebei/DF/2008(H9N2)-like-AIVs; this process was also completed as far back as 2013, but the endemic regions was only limited to the mainland China (Figs. 2 and 3, and Additional file 3: 2c, 3c, 7c, and 8c).

It is worth mentioning that the internal six segments for human infected AIVs H7N9 and H10N8 emerged in China during 2013–2014 were also originated from AIV H9N2. Reassortment likely happened between AIVs H5N1 and H9N2 also, and for example, the internal six segments of A/chicken/Fujian/FZ02/2011(H9N2) were similar with AIV H5N1 more than AIV H9N2 in the same period.

## Discussion

Since the first case of human infection with AIV H5N6 occurred in China in 2014, its origin has been an important topic. It is believed that the virus is a triple reassortant avian influenza virus containing an HA from clade 2.3.4.4 H5 viruses, a NA from H6N6 viruses, and 6 internal genes from clade 2.3.2.1 H5 viruses [7–9]. Our study however, found that concerning HA, NA, or 6 internal genes, human infected isolates of AIV H5N6 in China had other origins, and at least one strain of them had derived its 6 internal genes from AIV H9N2, which had circulated in Eastern China for a very long time. It is similar to AIVs H7N9 and H10N8, which emerged in China shortly before [1, 2]. Therefore, human infected AIV H5N6 in China during 2014–2016 is likely to have more complicated origin than it is generally believed.

As a segmented negative-strand RNA virus, influenza virus is characterized by a high frequency of reassortment. Reassortment can only occur among viruses, which replicate within the same cells. The prerequisite for reassortment is an individual host that is simultaneously infected with multiple divergent virus strains [10, 11].

Some regions of China have suitable conditions for the emergence of novel influenza viruses, for example, the first H9N2 low-pathogenicity avian influenza virus (LPAIV) and H5N1 high-pathogenicity avian influenza virus (HPAIV) were found in Guangdong province of Eastern China in 1994 and 1996, respectively [12, 13]. Poultry farms, unvaccinated or vaccinated, had high carrying rates of AIVs H9N2 or H5N1 and often caused sporadic outbreaks [14–16]. In Eastern and Southern China, the perennial positive rate of antibody against H9 fluctuated between 5.3 and 12.8 %, and the rate of AIV H9N2 isolating could reach as high as 9 % in poultry. However, there has been no obvious epidemic with mass poultry deaths [17, 18]. High prevalence of AIVs H9N2 and H5N1 infections in poultry could result in the simultaneous infection to an individual host with multiple divergent AIVs, and then novel AIVs could occurred through reassortment. Indeed, HPAIVs strains of H5N1, H5N2, H5N6, and H5N8 have been found in China since 2000 and widely circulate currently. They occasionally cause human infections, including at least six subtypes of AIVs (H5N1, H6N1, H7N9, H9N2, H10N8, and H5N6) [16, 19, 20].

All this implies that HA5 has not only a complex evolutionary ecology, but also has a high frequency of reassortment. Understanding of the origin of novel AIVs will help targeting for early detection and containment of these viruses. Furthermore, on the HA1/HA2 cleavage site AIV H5N6 has a motif of 321-PLREKRR/KR*GLF-332 with a typical feature of high pathogenicity [7, 9, 21–24]; it means that both infection with poultries and spillover to human sporadically, such as AIV H5N6 emerged in China during 2014–2015, are likely to cause serious consequences.

## Conclusions

This study presents detailed analyses on the characteristics of reassortment of human infected AIV H5N6 currently circulated in China. It confirmed that AIV H5N6 isolates from both human and poultry in China during 2014–2015 were very heterogeneous; not only H5N1, but also H9N2, were involved in the reassortment of AIV H5N6. Also, both these AIV H5N6 isolates from human and poultry and their reassortment donors had experienced complicated evolutionary history. Therefore, the surveillance for influenza itself and its hosts from the perspective of epidemiology, virology and ecology should be further strengthened, especially on AIVs H9N2 and H5N1.

## Additional files

Additional file 1: 1a–8a: Matrices of genomes involved in this study. 1a, PB2 (fas 4.73mb); 2a, PB1 (fas 4.72mb); 3a, PA (fas 4.45mb); 4a, HA (fas 1.88mb); 5a, NP (fas 3.26mb); 6a, NA (fas 1.62mb); 7a, MP (fas 2.33mb); 8a, NEP (fas 1.99mb). (ZIP 1 mb)
Additional file 2: 1b–8b: Large-scale phylogenetic trees involved in this study. 1b, PB2 (mts 17.0mb); 2b, PB1 (mts 16.8mb); 3b, PA (mts 16.7mb); 4b, HA (mts 4.90mb); 5b, NP (mts 17.5mb); 6b, NA (mts 4.62mb); 7b, MP (mts 18.7mb); 8b, NEP (mts 17.3mb). (ZIP 18 mb)
Additional file 3: 2c, 3c, 7c, and 8c: Four accuracy phylogenetic trees involved in this study. 2c, PB1 (pdf 29.7 kb); 3c, PA (pdf 29.7 kb); 7c, MP (pdf 29.6 kb); 8c, NEP (pdf 29.5 kb). 1c (PB2), 4c (HA), 5c (NP), and 6c (NA) were put into the main text as Fig. 1 and Fig. 2. (ZIP 106 kb)
