# Supplementary figures and images for: The complexity of human infected AIV H5N6 isolated from China
Source: BMC Infect Dis. 2016 Oct 25;16:600. doi: 10.1186/s12879-016-1932-1 (PMC5078974; doi:10.1186/s12879-016-1932-1)

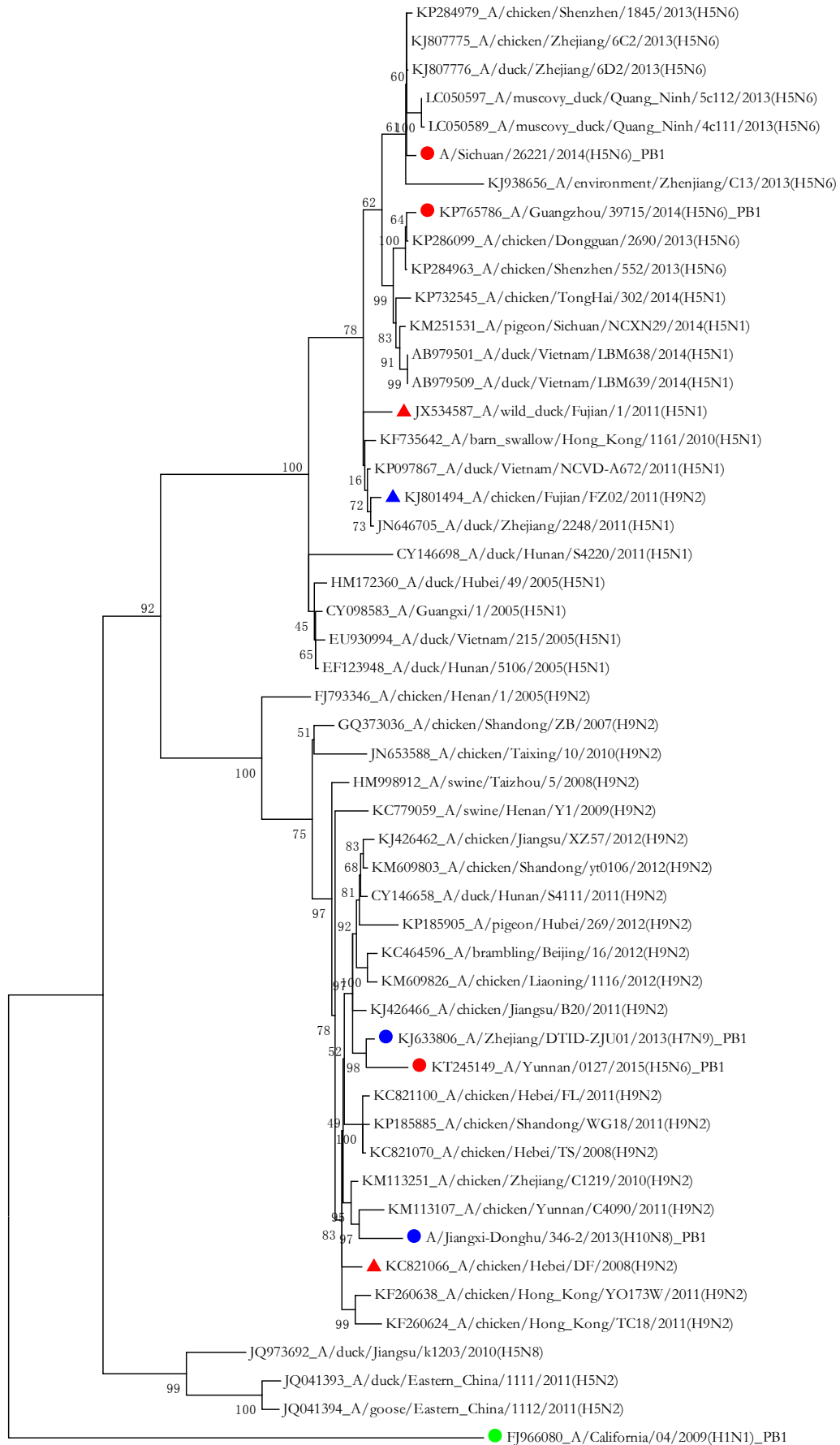

0.02

Supplement: Additional file 3: — 2c, 3c, 7c, and 8c: Four accuracy phylogenetic trees involved in this study. 2c, PB1 (pdf 29.7 kb); 3c, PA (pdf 29.7 kb); 7c, MP (pdf 29.6 kb); 8c, NEP (pdf 29.5 kb). 1c (PB2), 4c (HA), 5c (NP), and 6c (NA) were put into the main text as Fig. 1 and Fig. 2. (ZIP 106 kb) [file 12879_2016_1932_MOESM3_ESM.zip › Additional file 3/2c-PB1.pdf]

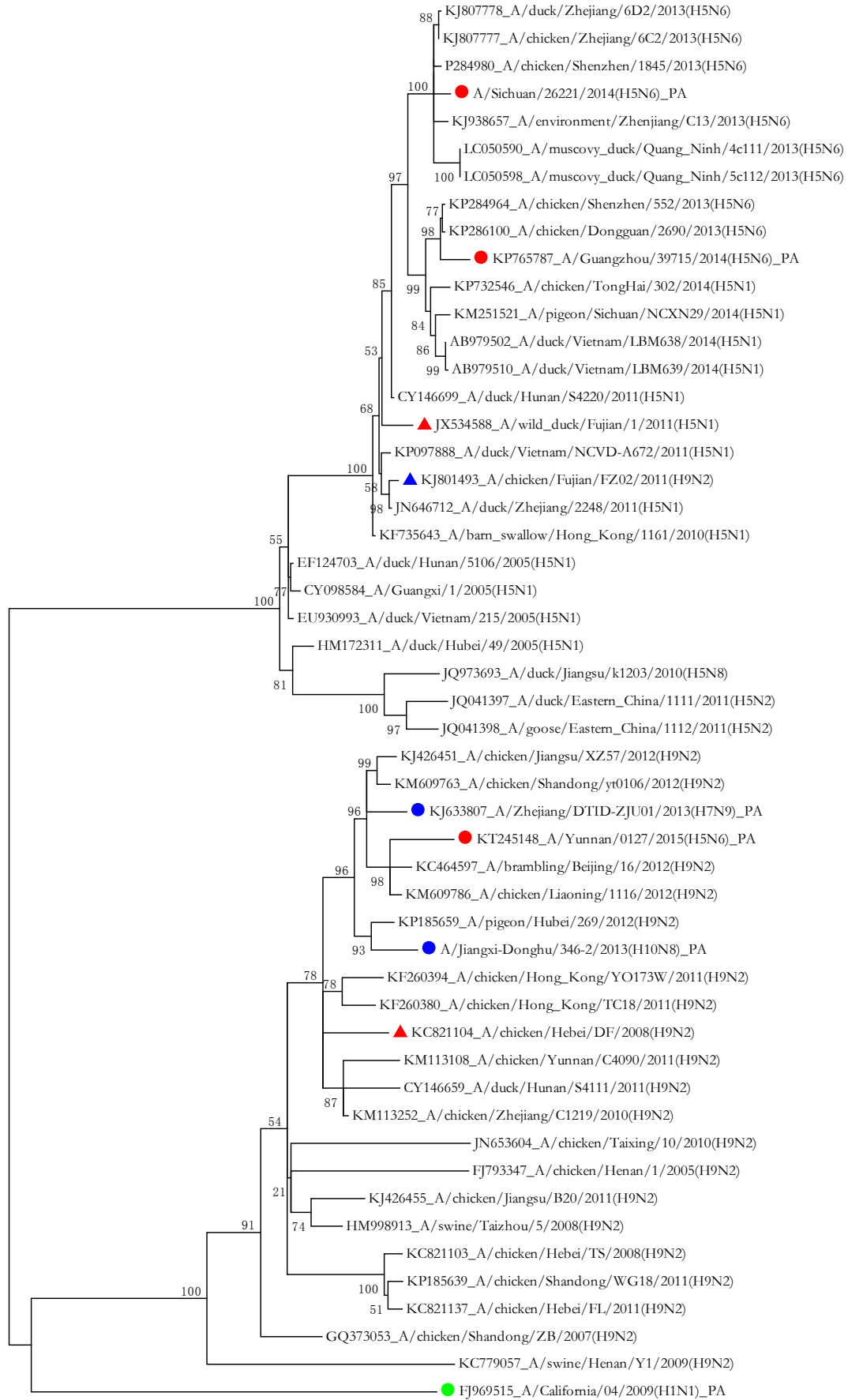

0.02

Supplement: Additional file 3: — 2c, 3c, 7c, and 8c: Four accuracy phylogenetic trees involved in this study. 2c, PB1 (pdf 29.7 kb); 3c, PA (pdf 29.7 kb); 7c, MP (pdf 29.6 kb); 8c, NEP (pdf 29.5 kb). 1c (PB2), 4c (HA), 5c (NP), and 6c (NA) were put into the main text as Fig. 1 and Fig. 2. (ZIP 106 kb) [file 12879_2016_1932_MOESM3_ESM.zip › Additional file 3/3c-PA.pdf]

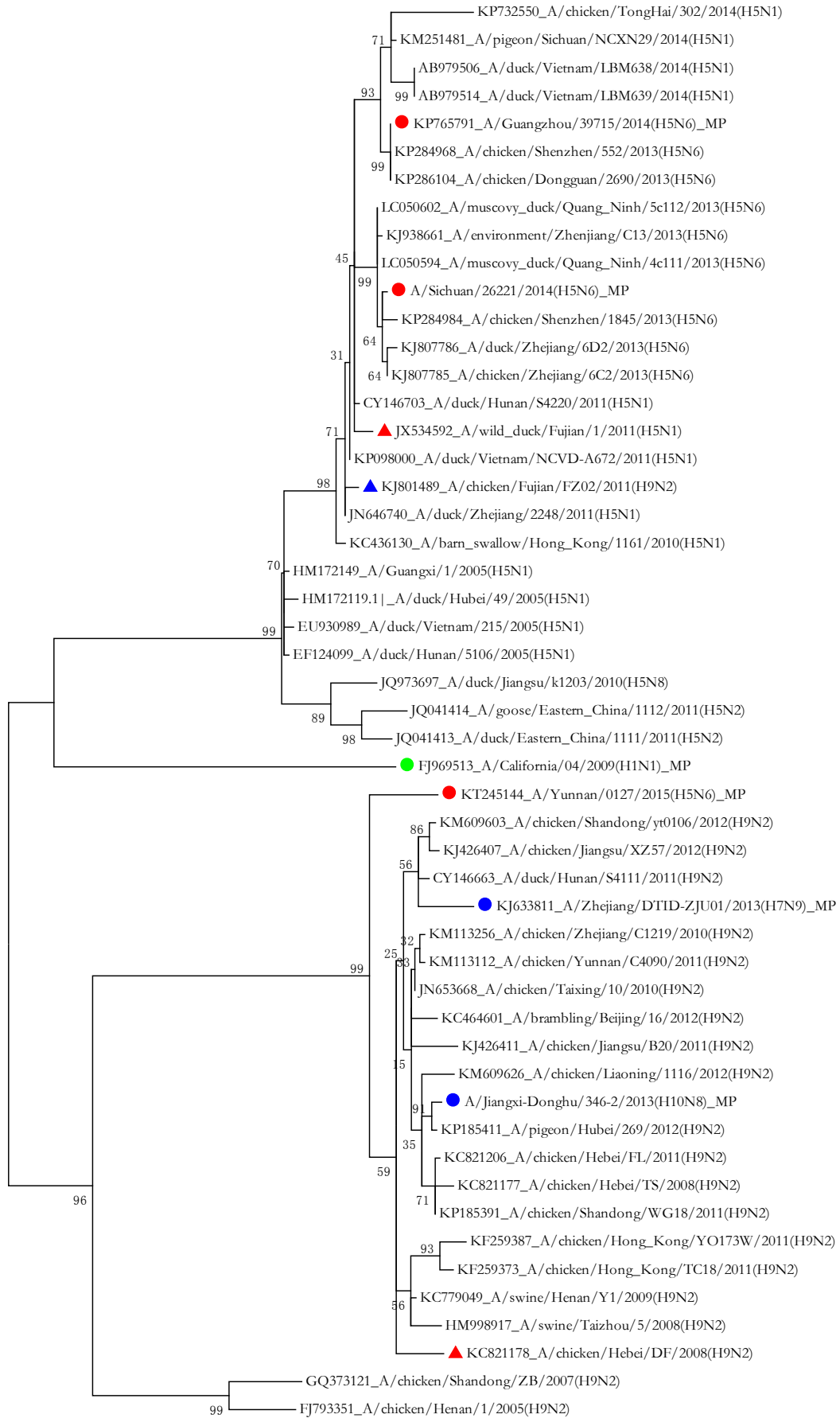

0.02

Supplement: Additional file 3: — 2c, 3c, 7c, and 8c: Four accuracy phylogenetic trees involved in this study. 2c, PB1 (pdf 29.7 kb); 3c, PA (pdf 29.7 kb); 7c, MP (pdf 29.6 kb); 8c, NEP (pdf 29.5 kb). 1c (PB2), 4c (HA), 5c (NP), and 6c (NA) were put into the main text as Fig. 1 and Fig. 2. (ZIP 106 kb) [file 12879_2016_1932_MOESM3_ESM.zip › Additional file 3/7c-MP.pdf]

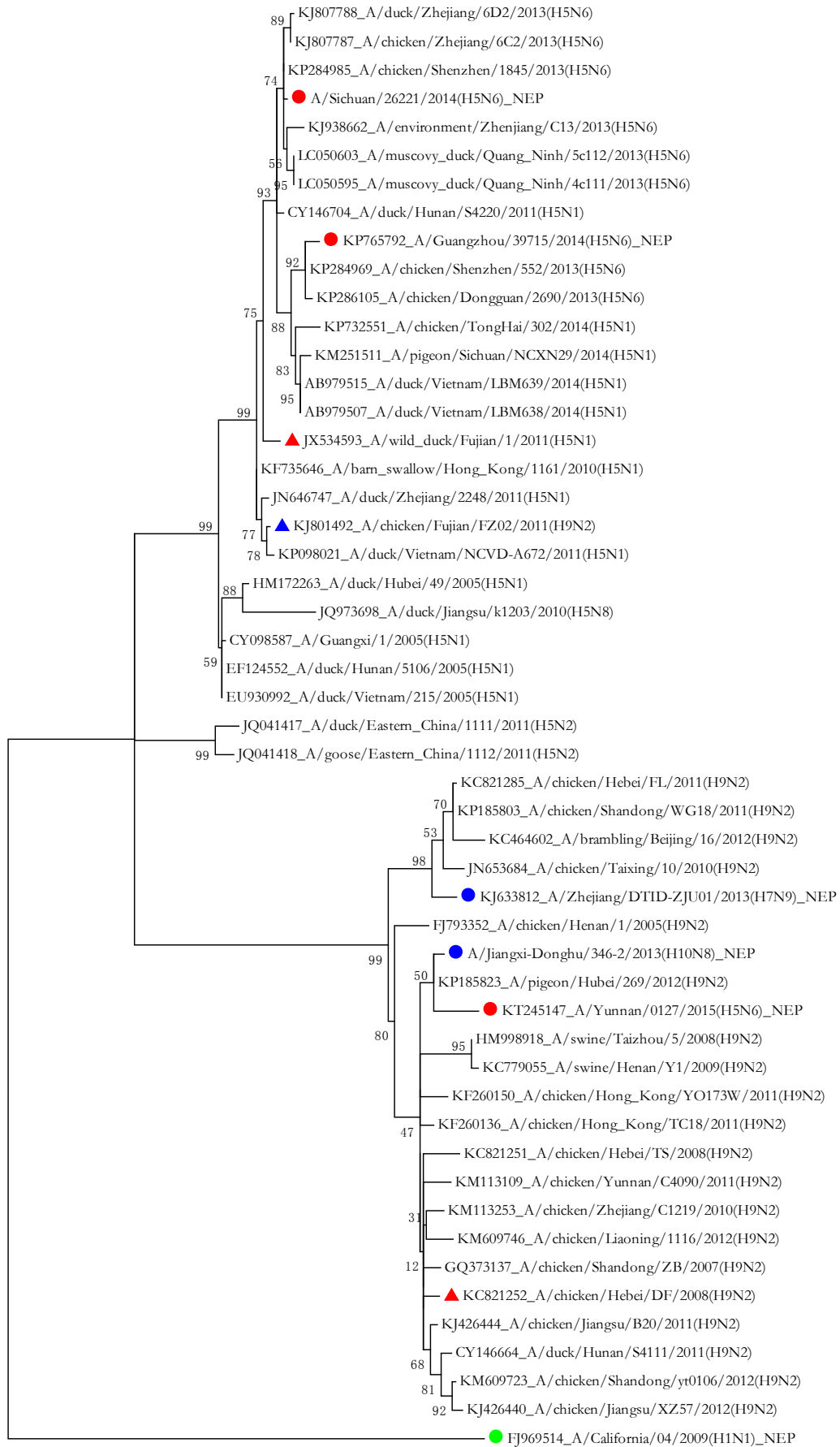

0.02

Supplement: Additional file 3: — 2c, 3c, 7c, and 8c: Four accuracy phylogenetic trees involved in this study. 2c, PB1 (pdf 29.7 kb); 3c, PA (pdf 29.7 kb); 7c, MP (pdf 29.6 kb); 8c, NEP (pdf 29.5 kb). 1c (PB2), 4c (HA), 5c (NP), and 6c (NA) were put into the main text as Fig. 1 and Fig. 2. (ZIP 106 kb) [file 12879_2016_1932_MOESM3_ESM.zip › Additional file 3/8c-NEP.pdf]
